# CHARACTERIZING NURSING HOME VISITATION POLICIES AND PROCESSES DURING THE COVID-19 PANDEMIC

**DOI:** 10.1093/geroni/igad104.3218

**Published:** 2023-12-21

**Authors:** Courtney Hawes, Joan Brazier, Amy Meehan, Emily Gabois

**Affiliations:** Brown University School of Public Health, Providence, Rhode Island, United States; Brown University School of Public Health, Providence, Rhode Island, United States; Brown University School of Public Health, Providence, Rhode Island, United States; Brown University School of Public Health, Providence, Rhode Island, United States

## Abstract

The COVID-19 pandemic drastically changed how nursing homes facilitated visitation between residents and their families. Nursing homes needed to modify visitation approaches in response to changing policies and guidelines from varying governing bodies. Additionally, restrictions were constantly adjusted due to fluctuations in COVID-19 infection rates within facilities and the larger communities in which they exist. Through data from 156 in-depth, semi-structured interviews with nursing home administrators, conducted at three-month intervals throughout the COVID-19 pandemic, administrator experiences are captured as they navigated these changes. Interviews provide valuable insight into strategies used during shut-down of in-person visitation, allowance of compassionate care and essential caregiver visits, and the resumption of in-person visitation, while also demonstrating the challenges of each visitation stage and the second-hand emotional responses of resident families. During the shut-down of in-person visitation, facilities conducted virtual visits and implemented new strategies including parades and window visits. Compassionate care and essential caregiver visits were used for those in hospice or experiencing weight loss and depression resulting from restrictions, though some administrators reported being flexible in their definitions of compassionate and essential caregiver visits. When in-person visitation resumed, interviews highlight the use of screening, social distancing, and monitoring during indoor visitation, as well as the approaches facilities took to respond to the challenges of staffing shortages and outbreak concerns. Study findings provide insight into how facilities may navigate future visitation changes and restrictions to maximize safety, the overall well-being of residents and the efficiency of facilitating visits.

